# Side Effects and Adverse Events After Treatment With Teprotumumab for Thyroid Eye Disease: A Retrospective Observational Case Series

**DOI:** 10.7759/cureus.58585

**Published:** 2024-04-19

**Authors:** Fabliha A Mukit, Andrew Manley, Akash B Patel, Marium Hashemi, Jacquelyn F Laplant, James C Fleming, Brian T Fowler

**Affiliations:** 1 Ophthalmology, Hamilton Eye Institute, Memphis, USA; 2 Ophthalmology, The University of Tennessee Health Science Center, Memphis, USA; 3 Internal Medicine, University of Texas Southwestern Medical Center, Dallas, USA; 4 Ophthalmic Plastic and Reconstructive Surgery, Medical College of Wisconsin, Milwaukee, USA; 5 Oculofacial Plastic and Reconstructive Surgery, Hamilton Eye Institute, Memphis, USA

**Keywords:** insulin-like growth factor 1 receptor (igf1-r), igf-1 receptor antagonist, teprotumumab, exophthalmos, thyroid eye disease

## Abstract

As the use of teprotumumab for thyroid eye disease (TED) becomes more prolific, there remains a scarcity of literature regarding the associated side effects and adverse events of teprotumumab use. The authors present a single-center retrospective, observational case review of TED patients who received at least a single dose of teprotumumab infusion at the oculofacial plastic surgery service between February 2020 and July 2023. The most predominant recollected side effects were fatigue, brittle nails, dry eye symptoms, hair loss, muscle spasms, and dry mouth. Significant adverse events were limited to two cases of a blood clot and a single case of pulmonary embolism. This is the first retrospective study of patient-reported side effects and adverse events experienced by a cohort of teprotumumab users.

## Introduction

Thyroid eye disease (TED), also known as Graves’ orbitopathy or thyroid-associated ophthalmopathy, is a rare and sight-threatening autoimmune disease affecting roughly 20-50 individuals per 100,000 annually [1]. It is the most common extra-thyroidal manifestation of Graves’ disease, and symptoms of TED tend to present within 18 months of its corresponding endocrine manifestations. Patients afflicted with TED can experience a heterogeneous constellation of symptoms resulting from the soft tissue changes that characterize the condition. Resultant symptoms of dry eyes, epiphora, and diplopia secondary to severe proptosis and complete vision loss all represent the spectrum of TED [1,2]. While the pathogenesis of TED continues to be investigated, the leading theories of pathogenesis are based on the complex interaction between circulating thyroid-stimulating hormone receptor antibodies (TRAb), the thyroid-stimulating hormone receptor (TSHR), and the insulin-like growth factor I receptor (IGF-1R) [3]. Activation of orbital fibroblasts in conjunction with B and T cells causes increased expression of extracellular matrix proteins and deposition of glycosaminoglycans into the soft tissues of the orbit, causing expansion [4,5]. The role of IGF-1R in this pathway has recently been identified and is the target of the newly developed TED medication on the market, teprotumumab.

In January 2020, the United States Food and Drug Administration (FDA) fast-tracked the use of teprotumumab for the treatment of active TED in adults [6]. Teprotumumab is a fully human monoclonal antibody that promotes internalization and degradation of the IGF-1R [7]. It has demonstrated significant results as a therapeutic agent in improving proptosis, diplopia, soft-tissue inflammation, and quality-of-life (QOL) in patients with TED [8,9]. However, given the ubiquity of IGF-1 signaling, systemic inhibition of these pathways is expected to produce a variety of adverse events [10]. The most commonly reported symptoms in the literature include muscle spasms, nausea, alopecia, diarrhea, fatigue, hearing impairment, and hyperglycemia. Evolving investigations now also report severe adverse events such as infusion reaction, pulmonary embolism, inflammatory bowel disease, and Hashimoto’s encephalopathy have recently been reported [8,9,11-13].

Due to its expedited FDA approval, our understanding of teprotumumab’s adverse event profile is actively being investigated and continues to change. Further understanding of the complete side effect profile is now even more important as the FDA recently expanded the patient profile to include chronic TED patients [14]. As the use of teprotumumab continues to increase in the TED patient population, so does our need to adequately investigate associated side effects and adverse events to provide patients with appropriate informed consent prior to initiating therapy. Notably, it is important to highlight that while hearing impairment was initially thought to affect roughly 10% of the drug’s users and be reversible upon cessation [8,9], new evidence suggests that up to 80% of individuals may experience this adverse event, with some reporting permanent hearing dysfunction [15]. This has prompted litigation against the drug’s manufacturers, Horizon Therapeutics plc (now acquired by Amgen Inc., California, United States), for failure to adequately document the drug’s risks [16]. Moreover, in a recent multi-center study, more than 80% of patients who received teprotumumab treatment experienced at least one teprotumumab-related adverse event, with most patients experiencing four or more, underscoring findings that adverse events are common with teprotumumab treatment for TED [13]. It is also important to note that the original randomized control trials on teprotumumab had narrow inclusion criteria that excluded many patients, such as those with chronic disease and uncontrolled diabetes [13], and thus may not be generalizable to the entire population of patients who are now eligible for teprotumumab treatment per FDA guidelines.

Here, we report on our single-center experience with teprotumumab infusions, utilizing clinical documentation and patient interviews to report the incidence of patient-recollected side effects and adverse events. This study looks at the presence or absence of all previously reported side effects or adverse events in our cohort of teprotumumab patients treated at a single university, as well as the side effect(s) that the patients perceived to be more significant from their treatment experience. Our study is the first perception study of the side effects and treatment experience of patients who underwent treatment with teprotumumab for active TED.

## Materials and methods

This is a single-center, retrospective, observational case series on teprotumumab-related side effects and adverse events that included patients with active TED who were undergoing or completed teprotumumab infusions between February 2020 and July 2023. The University of Tennessee Health Science Center Institutional Review Board approved the study (approval number: 22-08686-XP). This study adheres to the tenets of the Declaration of Helsinki as amended in 2013 and the Health Insurance Portability and Accountability Act. Informed consent was obtained from all patients.

All TED patients at the University of Tennessee’s Oculofacial Plastic and Reconstructive Surgery service were identified using related International Classification of Diseases, 10th Revision (ICD-10) codes of: E05.0 (thyrotoxicosis), E05.0 (thyrotoxicosis with diffuse goiter), E05.90 (hyperthyroidism), H06.2 (Graves’ ophthalmopathy), H05.20 (unspecified exophthalmos), H05.249 (constant exophthalmos, unspecified eye), H0.5.343 (constant exophthalmos, bilateral), and H05.241/H05.242 (constant exophthalmos right eye and left eye, respectively). After identifying all TED patients from the university practice, a retrospective chart review was completed to identify all patients with active TED who were planning to undergo treatment, were actively undergoing treatment, or had completed treatment with teprotumumab. All individuals over 18 years old who had completed at least one infusion of teprotumumab were included in our analysis. All patients followed the standard administration protocol of one infusion every three weeks, dosed at 10 mg/kg over 90 minutes for the first infusion, 20 mg/kg over 90 minutes for the second infusion, and then 20 mg/kg over 60 minutes for the remaining six infusions, for a total cycle involving eight infusions. Exclusion criteria were limited to any patient who did not undergo any teprotumumab infusions or did not return to the university practice. Objective information regarding the patient’s age, sex, gender, race, past medical history, history of previous thyroid treatments, Clinical Activity Score (CAS), number of teprotumumab infusions, and Hertel exophthalmometer measurements, both pre-infusion and post-infusion, and side effects were documented from the retrospective chart review. 

Due to restrictions imposed by the coronavirus disease 2019 (COVID-19) pandemic affecting patient follow-up and a non-standardized approach to questions addressed at follow-up visits, additional data regarding side effects and adverse events was collected through standardized phone interviews (see Appendix A). A comprehensive literature review of all teprotumumab-related papers published from October 2014 to August 2022 was conducted to identify all reported side effects and adverse events. Past medical history questions were developed to address any association with an increase in or new symptoms from teprotumumab infusions. During each interview, a standardized questionnaire was delivered to systematically address each patient’s relevant past medical history (diabetes, prior insulin use, hypertension, hearing loss, hearing device use, hair loss, muscle spasm, headache), side effects and adverse events associated with teprotumumab, resolution of side effects and adverse events, and improvement in TED-associated symptoms; it was also asked if the patient would recommend the use of teprotumumab. 

The collected data underwent descriptive analysis and logistic regression analysis. Patient characteristics were described using mean and standard deviation for the continuous variables and proportions were used for categorical variables. Categorical variables were also compared using Fisher’s test with contingency tables to estimate each historical condition’s predictive relationship with the new or increased side effect. Odds ratios with 95% confidence intervals and p-values were found. P-values <0.05 were considered statistically significant. All statistical analyses were conducted using R 4.2.0 (R Foundation for Statistical Computing, Vienna, Austria).

## Results

Thirty-eight patients were included in this study (Table 1). Of these patients, 35 were females (92.11%) and three were males (7.89%), with an average age of 58.2 years (standard deviation (SD) of 14.1, range of 30-85 years). Twenty patients identified as Caucasian (52.63%), 17 as African American (44.7%), and one as East-Asian (2.63%). Twenty-five patients completed the recommended eight infusion series, three patients participated in 16 infusions (second cycle), with the remaining 10 patients completing between one to seven infusions at the time of the interview. Ten patients (26.32%) had a prior surgical decompression and 11 patients (28.95%) had completed a trial of oral steroids prior to starting teprotumumab therapy. One patient declined to participate in the interview due to the psychological stress associated with teprotumumab infusions and two patients provided partial participation.

**Table 1:** Patient Characteristics

Characteristics	Number of Patients	Total Number of Patients (N)	Percentage (n/N x 100)
Race			
African American	17	38	44.74%
Caucasian	20	38	52.63%
Asian	1	38	2.63%
Gender			
Female	35	38	92.11%
Male	3	38	7.89%
Number of Infusions Completed			
1	1	38	2.63%
3	3	38	7.89%
5	1	38	2.63%
6	3	38	7.89%
7	2	38	5.26%
8	25	38	65.79%
16	3	38	7.89%
Other Thyroid Treatment			
Y	20	36	55.56%
N	16	36	44.44%
Surgical Decompression			
Y	10	38	26.32%
N	28	38	73.68%
Oral Steroids			
Y	11	38	28.95%
N	27	38	71.05%

Subjective symptoms and adverse events

There were six side effects experienced by the majority of the study population (Table 2). Twenty-four patients reported new or worsening fatigue (68.57%, n=35), 21 reported development of brittle nails (60%, n=35), 21 reported new or worsened dry eyes (60%), 19 reported new or worsened hair loss (54.29%, n=35), 18 reported new or worsening muscle spasms involving the extremities (51.43%, n=35), and 18 reported new or worsening dry mouth (51.43%, n=35). Two out of 35 patients experienced a blood clot (5.71%, n=35), and one was diagnosed with a pulmonary embolus (2.86%). Less than 40% of individuals experienced: new or worsened tinnitus (37.14%), new or increased hyperglycemia (34.29%), new or worsened gastrointestinal distress (34.29%), new or worsened dizziness (34.29%), new or worsened hearing loss (28.57%), altered appetite (28.57%), weight loss (22.86%), weight gain (22.86%), new or worsened headaches (14.29%), infusion site reactions (11.43%), increased insulin requirements (8.57%), urinary tract infection (8.57%), and need for a new endocrinologist (5.71%).

**Table 2:** Patient-Reported Side Effects and Adverse Events

Side Effect or Adverse Event	Number of Patients (n)	Total Number of Patients (N)	Percentage (n/N x 100)
Infusion Site Reaction	4	35	11.43%
New or Increased Muscle Spasm	18	35	51.43%
New or Increased Alopecia	19	35	54.29%
New or Increased Hyperglycemia	12	35	34.29%
New or Increased Use of Insulin	3	35	8.57%
New Referral to Endocrinologist	2	35	5.71%
New or Increased Gastrointestinal Distress	12	35	34.29%
New or Increased Fatigue	24	35	68.57%
New or Increased Headache	5	35	14.29%
New or Increased Subjective Hearing Loss	10	35	28.57%
New or Increased Tinnitus	13	35	37.14%
New or Increased Dizziness	12	35	34.29%
New or Increased Dry Eye Symptoms	21	35	60.00%
New or Increased Dry Mouth Symptoms	18	35	51.43%
Urinary Tract Infection Post-Infusion	3	35	8.57%
Weight Loss Post-Infusion	8	35	22.86%
Weight Gain Post-Infusion	8	35	22.86%
History of Blood Clot Post-Infusion	2	35	5.71%
History of Pulmonary Embolism Post-Infusion	1	35	2.86%
Altered Taste Post-Infusion	8	35	22.86%
Change in Appetite Post-Infusion	10	35	28.57%
New or Increased Brittle Nail	21	35	60.00%

Patient characteristics and risk factor evaluation

Relevant past medical history included six patients with a history of type 1 or type 2 diabetes mellitus (16.67%), three with a history of insulin use (8.33%), 19 with hypertension (52.78%) of which 15 had pressure between 110-135/70-85 (78.94%), eight with history of subjective hearing loss (22.22%), three with use of hearing loss devices (8.33%), 10 reported history of headaches (28.57%), 11 with history of hair loss (31.43%), and eight reported a history of muscle spasms (22.86%). There were no reports of a history of brittle nails (Table 3). 

**Table 3:** Patient Medical History

Medical Condition	Number of Patients (n)	Total Number of Patients (N)	Percentage (n/N x 100)
History of Diabetes (Type I or Type II)	6	36	16.67%
History of Insulin Use	3	36	8.33%
History of Hypertension (HTN)	19	36	52.78%
If yes to HTN, is your blood pressure in the normal range (110-135/70-85)?	15	19	78.94%
History of Hearing Loss	8	36	22.22%
If yes to hearing loss, do you use a hearing loss device?	3	36	8.33%
History of Headache	10	35	28.57%
History of Hair Loss	11	35	31.43%
History of Muscle Spams	8	35	22.86%
History of Brittle Nail	0	35	0.00%

Analysis using Fisher’s test with contingency tables was used to estimate each reported historical condition’s predictive relationship with a new or increased side effect (Table 4). A history of diabetes was found to predict new or increased hyperglycemia, with an odds ratio of 16.4 (p = 0.0082). There were no cases with a history of insulin use that required new or increased insulin. A comparison of a history of hearing loss to new or increasing subjective hearing loss had an odds ratio of 2.54 (p = 0.3482), and to new or increasing tinnitus had an odds ratio of 1.48 (p = 0.6769). A history of hair loss compared to new or increased hair loss had an odds ratio of 0.65 (p = 0.7166), a history of headache to new or increased headache had an odds ratio of 0.78 (p = 1.0000), and a history of muscle spasm to new or increased muscle spasm had an odds ratio of 1.42 (p = 1.000).

**Table 4:** Fischer’s Test with Contingency Table to Estimate Each Historical Condition’s Predictive Relationship to New or Increased Side Effects Post-Infusion

Variable	Odds Ratio	Lower Confidence Interval	Upper Confidence Interval	p-value
History of Diabetes compared to New or Increased Hyperglycemia (140mg/dl)	16.43	1.47	899.23	0.0082
History of Hearing Loss compared to New or Increased Subjective Hearing Loss	2.54	0.29	20.32	0.3482
History of Hearing Loss compared to New or Increased Tinnitus	1.48	0.18	11.00	0.06769
History of Hair Loss compared to New or Increased Hair Loss	0.65	0.12	3.42	0.7166
History of Headache compared to New or Increased Headache	0.78	0.01	11.39	1.0000
History of Muscle Spasm compared to New or Increased Muscle Spasm	1.42	0.20	11.62	1.0000

Symptom course and recommendations

Thirty-five of 38 patients (92.1%) reported subjective improvement of their thyroid eye disease (Table 5). Teprotumumab-associated symptoms were reported to have resolved in 28 out of 38 patients (73.68%) who completed therapy, with an average follow-up interview timeline of four weeks (range of one to 12 weeks) post last infusion. Thirty-one patients of 38 (81.58%) retrospectively recommended teprotumumab therapy and seven did not (18.42%). The spectrum of patient experience with teprotumumab ranged from the “best vision of their life” to triggering of subjective trauma from treatment resulting in an immediate cessation of the interview.

**Table 5:** Patient Symptom Course and Recommendations

Question	Number of Patients (n)	Total Number of Patients (N)	Percentage (n/N x 100)
Did Thyroid Eye Disease (TED) improve on Tepezza?	35	38	92.11%
Y	35	38	92.11%
N	3	38	7.89%
Did adverse symptoms resolve after completion of Tepezza treatment?			
Y	28	38	73.68%
N	10	38	26.32%
Would you recommend Tepezza?			
Y	31	38	81.58%
N	7	38	18.42%

## Discussion

Since its FDA approval in 2020, teprotumumab has emerged as a promising therapy for the management of TED. Patients undergoing treatment with the drug demonstrated lasting responses in proptosis, inflammation, diplopia, and QoL compared to placebo [8,9,17]. Additionally, when compared to other treatment modalities for TED (i.e., glucocorticoids, orbital radiotherapy, and other monoclonal antibodies), meta-analyses have demonstrated teprotumumab’s superiority in several of these measures [18-20].

Despite these benefits, patients may experience a wide range of adverse events associated with the drug’s use. Due to its fast-tracked FDA approval, few clinical trials were conducted on teprotumumab prior to its commercialization; thus, the adverse event profile remains incomplete. This knowledge gap impairs the informed consent process as patients and physicians may be unaware of the full spectrum of risks associated with the drug’s use. Moreover, prior to a recent large multicenter study, past clinical trials did not include patients with inactive TED, chronic TED, or comorbidities such as uncontrolled diabetes [13]. It is important to study this drug in a wider population of patients with TED who may be at increased risk for adverse events due to their comorbid health conditions [13]. The recent FDA approval for the use of teprotumumab for the treatment of all stages of TED, including inactive TED [14], further underscores the need to study adverse events in this group of patients. 

We conducted the present study to better characterize teprotumumab’s adverse event profile as well as to report the first patient perception study on teprotumumab use in a wider population of patients most recently approved to use teprotumumab by the FDA. In our analysis, we were able to provide information on the frequency of adverse events, persistence or resolution post treatment, and associated risk factors through a retrospective evaluation of teprotumumab patient experiences.

In our cohort, the most commonly patient-reported symptoms were fatigue (68.57%), brittle nails (60%), dry eye symptoms (60%), hair loss (54.29%), muscle spasms (51.43%), and dry mouth (51.43%). Less commonly, patients experienced infusion site reactions, hyperglycemia, increased insulin requirements, gastrointestinal distress, headaches, hearing loss, tinnitus, dizziness, urinary tract infections (UTIs), weight changes, and altered appetite.

Of the 12 patients who had new or increased hyperglycemia on daily blood glucose measurements, nine had minor adjustments to their non-insulin diabetic medication management, two required initiation of insulin, and one had an increase in insulin requirements. Of the two that required initiation of insulin, both had new referrals to endocrinology, and one had to stop treatment due to severe hyperglycemia and hospitalization. All patients who reported new or increased subjective hearing loss (10 patients) were referred to otolaryngology for further auditory assessment and testing. Of those 10 patients, eight had a known history of hearing loss and three used a hearing loss device prior to beginning teprotumumab. From our cohort, no patient was found to have objective hearing loss from testing by the otolaryngology service. 

Notably, two of our patients developed new lower extremity blood clots (5.9%), after the third and fifth infusion, respectively, with one experiencing a pulmonary embolus, following initiation of teprotumumab. Further medical history review of these two patients revealed other confounding comorbidities, and a history of previous deep vein thrombosis prior to starting teprotumumab. Most of these adverse events have been previously documented in clinical trials and case reports; however, the findings of UTIs, blood clots, and pulmonary embolus appear to be unique to the current study and further studies are warranted to investigate whether this could be related to the therapy itself [8,9,21-23].

Of the 38 patients interviewed, 35 reported subjective improvements in TED symptoms. The retrospective chart review showed all 38 patients interviewed had at least 2 mm improvement on pre- and post-infusion Hertel exophthalmometry. In addition, teprotumumab-associated side effects were subjectively resolved in 28 of the 38 patients. A total of 31 patients stated that they would recommend treatment with teprotumumab for TED, indicating that the drug was generally well-tolerated in our cohort. Serious adverse events did occur in some patients, leading to the discontinuation of the drug and an unwillingness to recommend its use. In these patients, the symptomatic improvements were incongruent with the risks associated with the drug. One patient developed severe hyperglycemia that led to the cessation of her treatment, while another was unable to share her reaction to the drug because of the severe emotional distress it has caused. Although rare, serious adverse events such as those documented in the current study are important to consider when initiating teprotumumab. Other previously reported severe reactions include the onset of inflammatory bowel disease, Hashimoto’s encephalopathy, and *Escherichia coli *sepsis [9,11-13].

When evaluating for associated risk factors with these adverse events, we found that the development of hyperglycemia following teprotumumab initiation was associated with a history of diabetes, in accordance with prior studies. Reports of hearing loss, tinnitus, and muscle spasms were also associated with increased odds of experiencing new or worsened hearing loss, tinnitus, and muscle spasms, respectively; however, these results did not achieve statistical significance. Additionally, a history of hair loss and headaches were associated with decreased odds of these adverse events following teprotumumab initiation, which also failed to meet statistical significance. This incongruence and lack of statistical significance is likely impacted by the study’s small patient cohort and resultant low power. However, these results may suggest that teprotumumab’s adverse events arise from its interaction with ongoing, physiologically impaired processes. The increased risk of hearing loss following teprotumumab treatment in patients with a history of hearing loss did show a significant correlation in other studies [13]. These risk factors were not reported in the initial randomized control trials on teprotumumab and, thus, introduced new information that has the potential to guide recognition and minimization of these adverse effects by influencing patient selection, baseline screening, and monitoring through the treatment course. Further studies are warranted to explore these risk factors and interventions further.

While the pathogenesis of the adverse events experienced by teprotumumab users is multifactorial, many are likely related to the drug’s actions on IGF-1 signaling pathways. IGF-1 receptors are ubiquitously expressed throughout the body and are responsible for a wide range of biological activities [24]. Metabolically, IGF-1 has been shown to decrease growth hormone signaling and consequently modulate glucose utilization, mobilization, and insulin sensitivity in adipocytes and skeletal muscles [24,25]. Within the vasculature, IGF-1 is partly responsible for endothelial cells’ anti-thrombotic properties by increasing potassium channel conductance and production of nitric oxide (NO), which induces vasorelaxation and decreases platelet aggregation [26]. Most notable, however, is the hormone’s role in growth and development. Transgenic and knockout mice models of IGF-1 or IGF-1R deficiency have demonstrated the importance of this signaling pathway on somatic growth. Organisms lacking these tend to experience significant growth restrictions [27], a phenotype that is similarly observed in individuals with pathogenic variants of IGF-1 or IGF-1R [28]. IGF-1’s autocrine functions have also been implicated in the normal development and proliferation of epidermal tissues, enterocytes, and cochlear cells [29-31]. Thus, it is reasonable to anticipate adverse events such as glucose dysregulation, blood clots, muscle spasms, hearing loss, hair loss, and gastrointestinal distress in individuals on teprotumumab.

Limitations of the current study include its retrospective nature, review of non-standardized charts, and reliance on patient interviews for data collection. Many individuals completed teprotumumab years prior to our study, increasing the risk of recall bias in comparison to immediate data collection from follow-ups. However, this does provide a benefit in that the effects reported were likely those that affected the patients most significantly. However, this method of data collection allowed patients to share the side effects that affected them the most after treatment. Moreover, it is also the first study to ask patients whether they would still recommend treatment with teprotumumab. Additionally, our study population included only individuals treated at a single center from a single region of the United States and did not enroll a significant number of males. Although this latter fact may be reflective of the female predominance of autoimmune conditions, this introduces the potential for selection bias.

## Conclusions

Our study demonstrates that most patients treated with teprotumumab for TED experienced at least one side effect. Importantly, many of these were shown to resolve upon the drug’s cessation and rarely impacted patients’ subjective experiences with the drug, as indicated by their willingness to recommend its use. Understanding a drug’s risk profile is equally as important as understanding its efficacy. Our inclusion of patients with long-standing disease and comorbid conditions further allowed us to study a wider population of patients based on the FDA’s most recent eligibility criteria. This also enabled us to study any association the use of teprotumumab may have with other comorbid conditions like diabetes, which would provide more guidance on how to screen, monitor, and manage patients who have more conditions in addition to their TED. 

## Appendices

Appendix A: phone interview questionnaire

Name:                                        

DOB:

Number of infusions completed at the time of the interview:

Patient Medical History

1.    DM - Type 1 or Type 2 - Yes/No

       Insulin Use? 

2.    HTN - Yes/No

       Controlled or Uncontrolled?

3.    Hx of Hearing Loss - Yes/No

       Subjective or objective (tested)?

       Any hearing device?

4.    Hx of Headaches - Yes/No

5.    Hx of Hair Loss - Yes/No

6.    Hx of Muscle Spasm - Yes/No

Side Effects and Adverse Events

For all questions, ask if they recall when it happened (after which infusion). Did it cause them to stop infusions? If infusions stopped or were completed, did symptoms resolve?

1.     Development of Infusion site rash - Yes/No

        Location(s) of rash:

2.     Increased Muscle Spasm - Yes/No

        Location(s):

3.     Increased Hair loss - Yes/No

4.     Increased Hyperglycemia (BG range, A1c, med regimen, endocrinologist)

        Did blood glucose range increase at any time above 140mg/dl - Yes/No

        Increase in A1c after starting Teprotumumab? Yes/No

        Increased Insulin Use - Yes/No

        New referral to Endocrinologist - Yes/No

5.     Increased GI distress (Diarrhea, Bloating, Abdominal Pain, Constipation, Hematochezia, Melena) - Yes/No

6.     Increased Fatigue - Yes/No

7.      Increased Headache - Yes/No

8.     Increased Hearing Loss - Yes/No

9.     Increased Tinnitus - Yes/No

10.   Increased Vertigo/Dizziness - Yes/No

11.    Increased Dry Eyes - Yes/No

12.   Increased Dry Mouth - Yes/No

13.   Any occurrence of a UTI - Yes/No

14.   Any Weight Loss - Yes/No

15.   Any Weight Gain - Yes/No

16.   Any Blood Clot - Yes/No

17.   Any Pulmonary embolus - Yes/No

18.   Any Altered Taste- Yes/No

19.   Any Change in Appetite - Yes/No

20.   Any occurrence of Brittle Nails - Yes/No

21.   Other symptoms:

22.  At this time, would you recommend Teprotumumab?  - Yes/No
